# (Pro)renin receptor involves in myocardial fibrosis and oxidative stress in diabetic cardiomyopathy via the PRR–YAP pathway

**DOI:** 10.1038/s41598-021-82776-2

**Published:** 2021-02-05

**Authors:** Shiran Yu, Xuefei Dong, Min Yang, Qingtao Yu, Jie Xiong, Jing Chen, Bo Dong, Qing Su

**Affiliations:** 1 Department of Cardiology, Shandong Provincial Hospital, Cheeloo College of Medicine, Shandong University, Jinan, 250021 Shandong China; 2 University of Hull, Hull, UK; 3 Department of Laboratory, The Third Hospital of Jinan, Jinan, China; 4 Department of Cardiology, Center for Cardiovascular Translational Research, Beijing Key Laboratory of Early Prediction and Intervention of Acute Myocardial Infarction, Peking University People’s Hospital, Beijing, 100044 China; 5 Warwick Medical School, University of Warwick, Coventry, UK; 6 Jining Medical University, Jining, China; 7 The Key Laboratory of Cardiovascular Remodeling and Function Research, Chinese Ministry of Education, Qilu Hospital of Shandong University, Jinan, 250012 China; 8 Department of Cardiology, Shandong Provincial Hospital Affiliated to Shandong First Medical University, Jinan, China; 9 Department of Endocrinology, Xinhua Hospital, Shanghai Jiaotong University School of Medicine, Shanghai, 200092 China

**Keywords:** Cardiology, Endocrinology, Medical research, Pathogenesis

## Abstract

(Pro)renin receptor (PRR) and Yes-associated protein (YAP) play an important role in cardiovascular diseases. However, the role of PRR–YAP pathway in the pathogenesis of DCM is also not clear. We hypothesized that PRR–YAP pathway may promote pathological injuries in DCM by triggering redox. Wistar rats and neonatal rat cardiac fibroblasts were respectively used in vivo and in vitro studies. In order to observe the effects of PRR mediated YAP pathway on the pathogenesis of DCM, animal experiments were divided into 3 parts, including the evaluation the effects of PRR overexpression, PRR RNAi silencing and YAP RNAi silencing. Recombinant-adenoviruses-carried-PRR-gene (Ad-PRR), Ad-PRR-shRNA and lentivirus-carried-YAP-shRNA were constructed and the effects of PRR mediated YAP on the pathogenesis of DCM were evaluated. YAP specific inhibitor Verteporfin was also administrated in cardiac fibroblasts to explore the impact of PRR–YAP pathway on oxidative stress and myocardial fibrosis. The results displayed that PRR overexpression could enhance YAP expression but PRR RNAi silencing down-regulated its expression. Moreover, PRR overexpression could exacerbate oxidative stress and myocardial fibrosis in DCM, and these pathological changes could be rescued by YAP blockade. We concluded that PRR–YAP pathway plays a key role in the pathogenesis of DCM.

## Introduction

Diabetic cardiomyopathy (DCM) is a serious cardiovascular complication of diabetes mellitus, and it is tightly associated with heart failure and cardiovascular mortality independent on other cardiac risk factors, such as coronary artery disease and hypertension. Although there are some possible therapeutic approaches for DCM, these approaches result in imperfect outcomes. Therefore, it is necessary to better understand the fundamental molecular abnormalities in DCM and explore new therapeutic targets^1^. DCM is characterized by several abnormalities in structure and function that can exacerbate cardiac dysfunction, and it also features a series of pathological progresses including inflammation, oxidative stress, apoptosis and myocardial fibrosis that finally result in cardiac remodeling and can even lead to heart failure^2^. It is reported that the excessive activation of both the systemic and cardiac tissue renin-angiotensin system (RAS) exacerbates the development of DCM, and inhibitors of the RAS, including angiotensin-converting enzyme inhibitors (ACEIs) and angiotensin receptor blockers (ARBs), are widely used in clinical treatment, but the morbidity and mortality of diabetic cardiovascular complications remain high^3^.

The (pro)renin receptor (PRR) is a novel component of the RAS is widely expressed in various organs such as the heart, kidneys and brain and involves in several pathological progresses of cardiovascular disease^4^.

It has been reported that the PRR is a multifunctional receptor that binds with renin or prorenin not only to accelerate Ang II production but also to directly activate other related pathological signaling pathways such as the MAPK pathway and TGF-β pathway, in which the level of Ang II is not changed but tightly associated with fibrosis, inflammation and cell proliferation^5^. It is also reported that PRR exhibits v-ATPase activity and activates canonical as well as non-canonical Wnt pathways playing an essential role in maintaining tissues homeostasis. *G*iven to this function if PRR totally knockout in embryonic or developmental period, it will lead to death. However, in pathological conditions such as diabetes mellitus, PRR primarily works as to enhance the RAS or MAPK signaling in addition to acting on V-ATPase. Meanwhile, increased V-ATPase activity in turn accelerates PRR transportation to the cell membrane and subsequently activates intracellular pathways, such as the MAPK pathway, involving in pathological progression^6,7^.

An existing study showed that blocking the PRR could reverse some pathological processes related to different cardiovascular diseases. A previous study demonstrated that PRR blockade could yield benefits in both spontaneously hypertensive rats (SHR) and myocardial infarction mice^5,8^. A recent report displayed that PRR expression is significantly increased in DCM^9^, which highlighted the importance of the PRR in the development of DCM. However, the effect of the PRR in DCM is not yet clear. Yes-associated protein (YAP) is well known as a central transcriptional effector of the highly conserved Hippo pathway and is involved in many diseases including cancer and cardiovascular diseases^10^. As an important transcriptional cofactor, YAP nuclear localization and activation could enhance the transcriptional activities of several functional proteins, such as the TEA domain transcription factor (TEAD) and Drosophila mothers against decapentaplegic protein (Smad), and it is involved in a series of pathological progresses like proliferation, oxidative stress and fibrosis^11,12^. It is reported that YAP activation is mediated by multiple pathways. The key active effector of RAS, angiotensin II (Ang II) is one of the most important mechanisms. Previous study showed that Ang II inhibits Hippo signaling and then stimulates YAP nuclear accumulation and activation in HEK293 cells^13^. In contrast, some reports found that ARBs could attenuate YAP activation^14^. A recent study showed that YAP expression could be increased and activated under high glucose conditions in the kidney and take part in kidney injury^15^. However, the change and effect of YAP in diabetic cardiomyopathy have not been explored.

To explore the effects of the PRR on YAP activation and observe whether the PRR mediates YAP pathway involvement in the pathogenesis of DCM, recombinant-adenoviruses-carried-PRR-gene (Ad-PRR), recombinant-adenoviruses-carried-PRR-shRNA (Ad-PRR-shRNA) and lentivirus-carried-YAP-shRNA (LV-YAP-shRNA) were constructed. The animal experiments were divided into 3 parts, including the evaluation of the effects of PRR overexpression, the assessment of the effects of PRR RNAi silencing and the evaluation of the effects of YAP RNAi silencing. The effects of PRR-mediated YAP on the pathogenesis of DCM were evaluated. We also used the YAP specific inhibitor Verteporfin in vitro and explored whether YAP blockade could reverse the effects of the PRR. It is reported that Verteporfin administration could inhibit YAP induced proliferation and other functions. We respectively evaluated each group’s oxidative stress level by detecting the SOD content, MDA content and activity of NADPH oxidase and assessed the level of myocardial fibrosis in DCM, including the expression of Smad, CTGF, collagen I and fibronectin in vivo and in vitro. In addition, the possible mechanism of the role of the PRR–YAP pathway in DCM was explored.

## Results

### PRR and YAP protein expression in DCM rats

**Figure 1:**
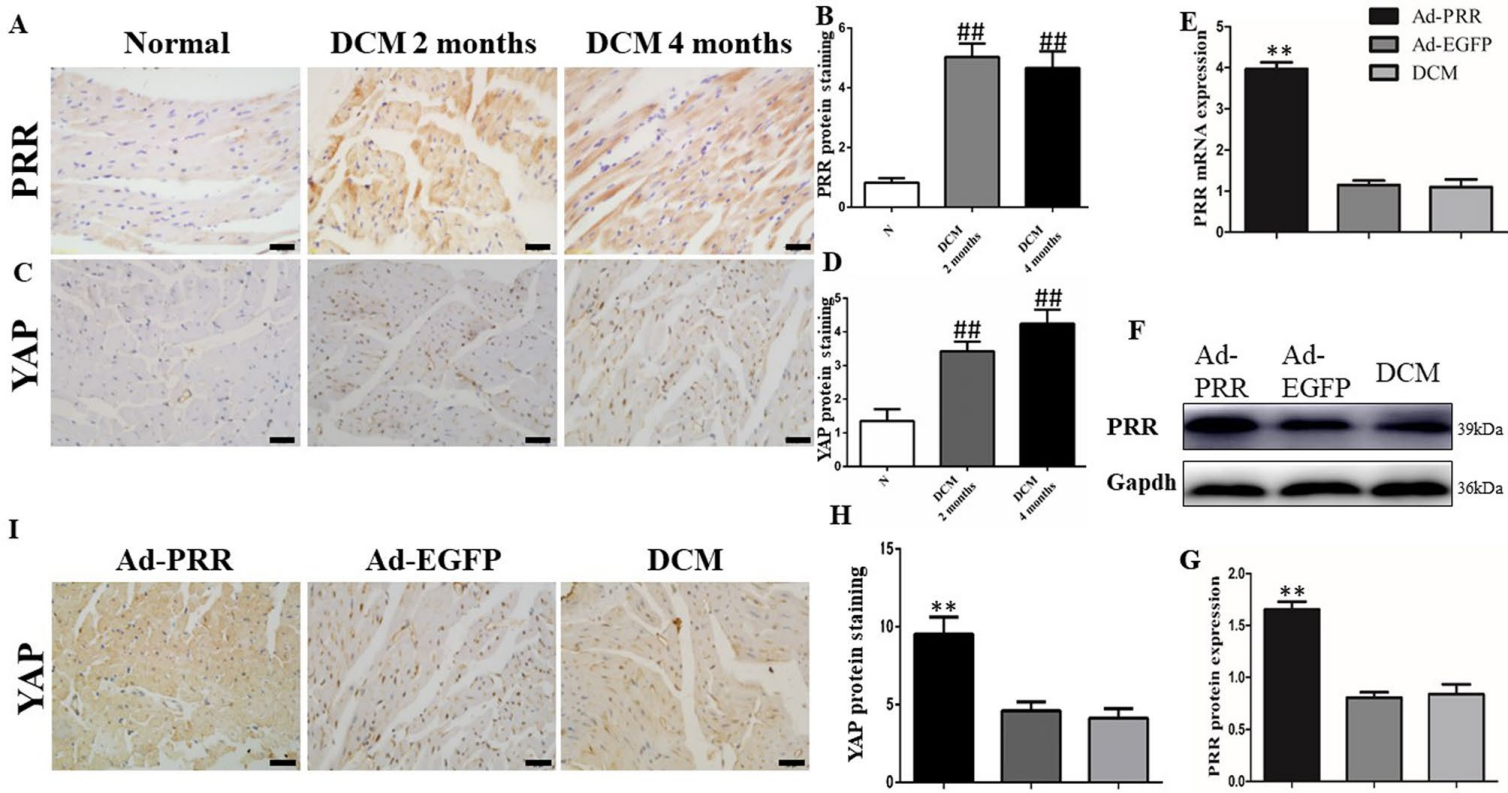
(Pro)renin receptor and YAP expression in rats. (**A**) Representative immunostaining of PRR protein expression 2 weeks and 4 weeks after STZ intraperitoneal injection. (**B**) Quantitative analysis of the results shown in (**A**). (**C**) Representative immunostaining of YAP protein expression 2 weeks and 4 weeks after STZ intraperitoneal injection. The results are calculated by counting positive expression area intensity and totally available tissue area size, and then the final result is the ratio of positive area intensity and totally available tissue area size. (**D**) Quantitative analysis of the results shown in (**C**). (**E**) PRR mRNA expression. (**F**) PRR protein expression in the 3 different groups. Figure (**F**) is cropped picture, omitting the repeated blots from the same group. The full-length blots/gel is complemented in supplementary figure 1. (**G**) Quantitative analysis of the results shown in (**F**). (**I**) Representative immunostaining of YAP protein expression in the 3 groups. (**H**) Quantitative analysis of the results shown in (**I**). (scale bar = 20 μm, magnification × 40). **p < 0.01 compared with the DCM group and Ad-EGFP group; ^##^*p* < 0.01 compared with the control group. In this part, each group had n = 20 rats at beginning, in 2 weeks took 8 rats from each group for transfection efficiency analysis. In 4 weeks after removing dead rats, the other of rats incorporate into analysis, in DCM group had 11 rats, Ad-PRR group had 11 rats and Ad-EGFP group had 12 rats finally.

PRR protein expression in DCM group rats and control group rats was detected. The results of immunohistochemistry showed that the PRR protein expression in DCM rats was much higher than that in control group rats both at 2 months and 4 months after STZ injection, (Fig. 1A,B, G, *p* < 0.01). Furthermore, the YAP protein expression levels in rat myocardial tissues both 2 months and 4 months after STZ injection were significantly increased compared with those in control group (Fig. 1C,D, *p* < 0.01).

At the end of experiment, the results of PCR showed that PRR mRNA expression in the Ad-PRR group was much higher than that in DCM group and Ad-EGFP group. The results of Western blot also showed the same result that PRR protein expression in the Ad-PRR group was significantly upregulated compared with that in the DCM group and Ad-EGFP group (Fig. 1E,F, *p* < 0.01).

After PRR overexpression, the results of immunohistochemistry showed that the YAP protein expression level in Ad-PRR group was much higher than that in the DCM group and Ad-EGFP group (Fig. 1H,I, *p* < 0.01).

### PRR overexpression increased TGF-β, CTGF and fibronectin expression in DCM rats

**Figure 2:**
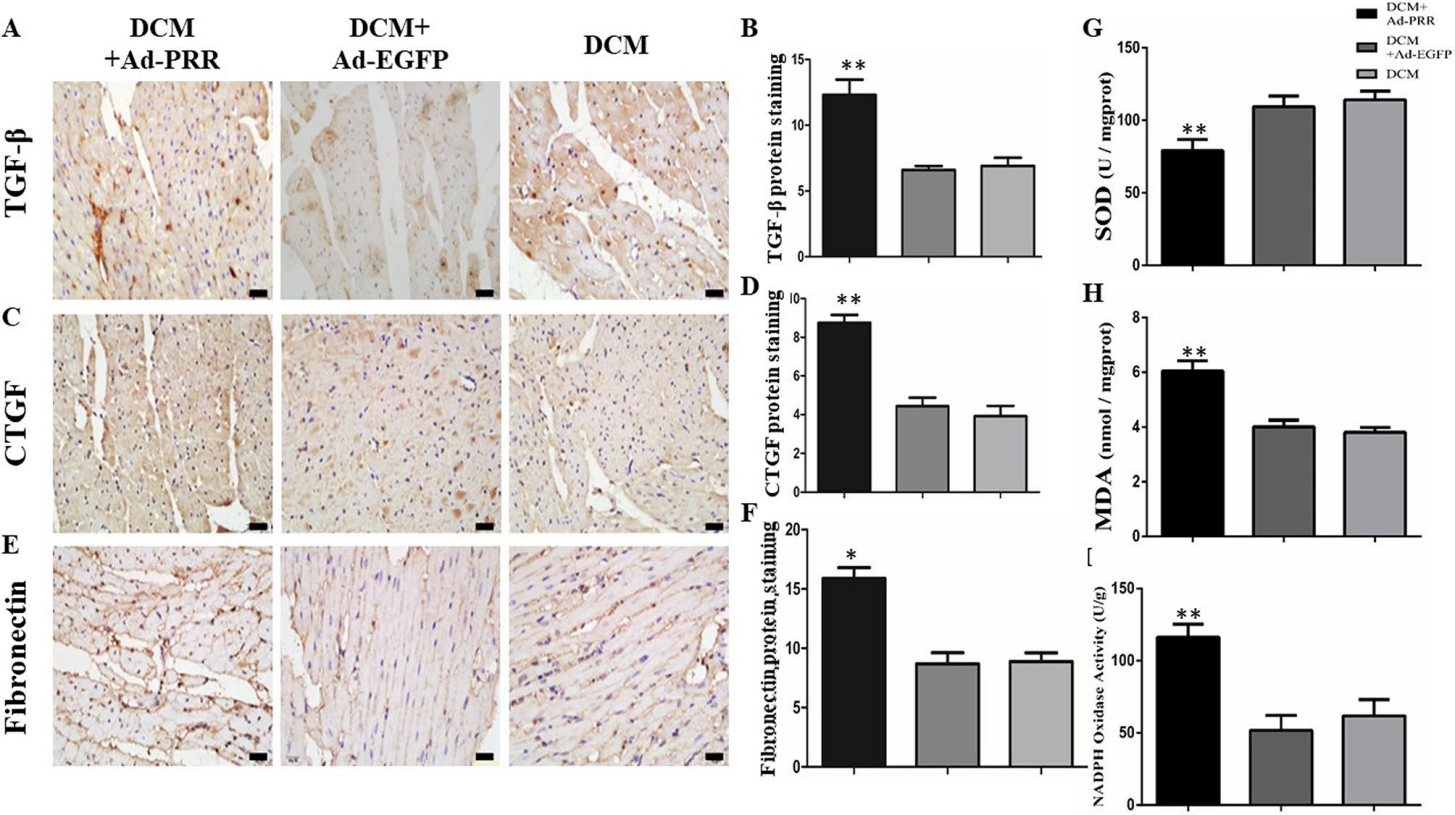
TGF-β, CTGF and fibronectin expression in the 3 groups and SOD and, MDA levels and NADPH oxidase activity in the 3 groups. (**A**) Immunostaining of TGF-β, (**B**) Quantitative analysis of the results shown in (**A**). (**C**) Immunostaining of CTGF, (**D**) Quantitative analysis of the results shown in (**C**). (**E**) Immunostaining of fibronectin. (**F**) Quantitative analysis of the results shown in (**E**). (**G**) SOD levels in the 3 groups. (**H**) MDA levels in the 3 groups. (**I**) NADPH oxidase activity in the DCM group, Ad-EGFP group, and Ad-PRR group. *p < 0.05, **p < 0.01 compared with the DCM group and Ad-EGFP group. Scale bar = 20 μm, magnification × 40. In 4 weeks after removing dead rats, the other of rats incorporate into analysis, in DCM group had 11 rats, Ad-PRR group had 11 rats and Ad-EGFP group had 12 rats finally.

The results of immunohistochemistry showed that TGF-β protein expression in the Ad-PRR group was significantly higher than that in the Ad-EGFP group and DCM group (Fig. 2A,B, *p* < 0.01). Similarly, CTGF protein expression in the Ad-PRR group was much higher than that in the DCM group and Ad-EGFP group (Fig. 2C,D, *p* < 0.01). For fibronectin protein expression, the results of immunohistochemistry showed that fibronectin protein expression was statistically higher in the Ad-PRR group than in the DCM group and Ad-EGFP group (Fig. 2E,F, *p* < 0.05).

### PRR is involved in oxidative stress responses in DCM rats

The SOD and MDA expression levels were observed, and the results showed that the SOD levels in the Ad-PRR group were much lower than those in the DCM group and Ad-EGFP group (Fig. 2G, *p* < 0.01). In contrast, compared with the DCM group and Ad-EGFP group, the MDA level was significantly increased in the Ad-PRR group (Fig. 2H, *p* < 0.01).

In addition, the activity of NADPH oxidase in each group was evaluated. The results displayed that NADPH oxidase activity was much higher in the Ad-PRR group than in the DCM group and Ad-EGFP group (Fig. 2I, *p* < 0.01).

### PRR and YAP protein expression in vitro

**Figure 3:**
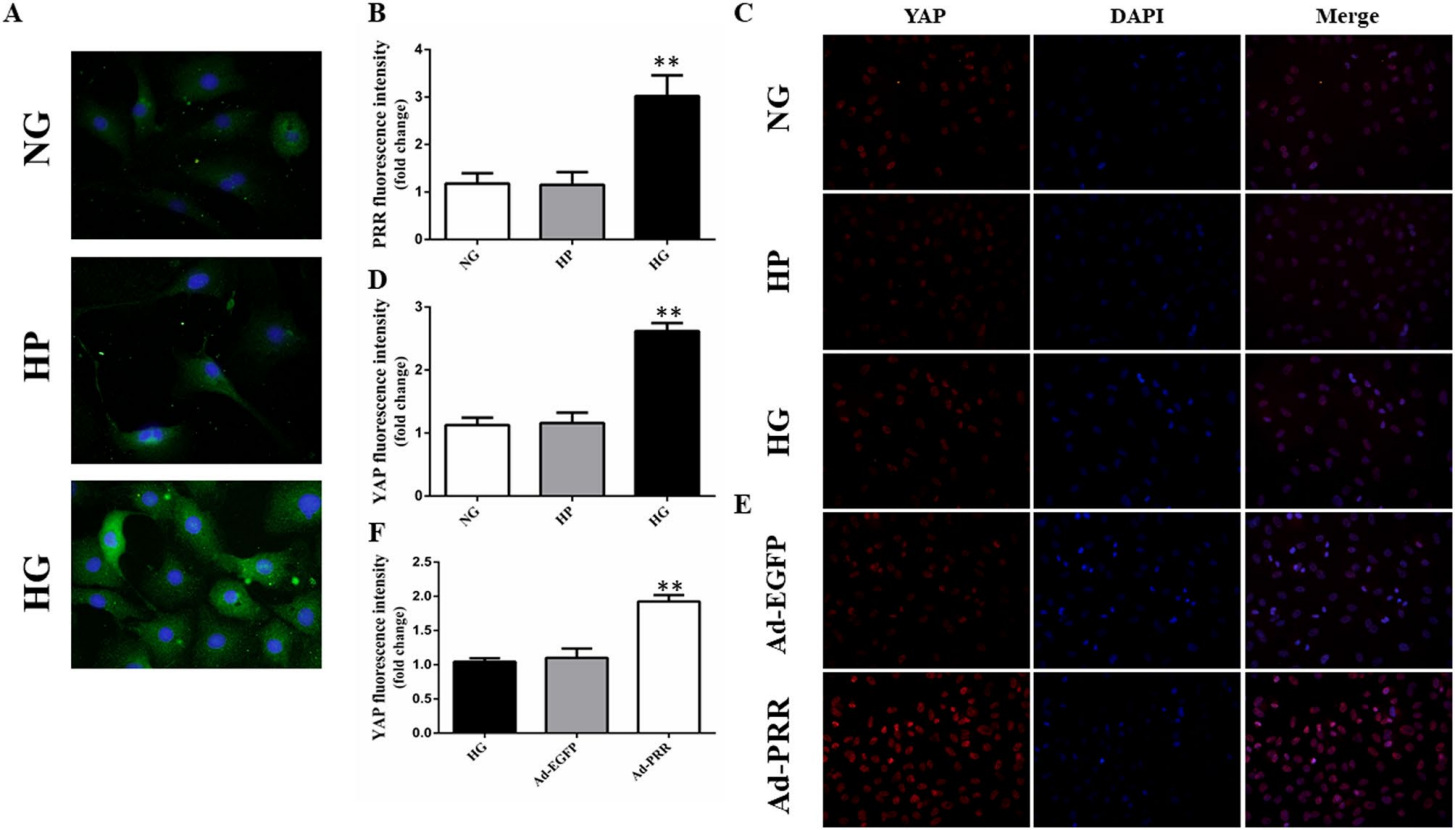
(Pro)renin receptor and YAP expression in cardiac fibroblasts. (**A**) Immunofluorescence showing PRR protein expression in the cardiac fibroblasts (CFs) in the control group, high permeability group and high glucose group. (**B**) Quantitative analysis of the results shown in (**A**). (**C**) Immunofluorescence showing YAP protein expression in the cardiac fibroblasts (CFs) in the control group, high permeability group and high glucose group. (**D**) Quantitative analysis of the results shown in (**C**). (**E**) Immunofluorescence showing YAP protein expression in the cardiac fibroblasts (CFs) in the high glucose group, Ad-EGFP group, and Ad-PRR group. (**F**) Quantitative analysis of the results shown in (**E**). **p < 0.01 compared with the control group and high permeability group. ##*p* < 0.01 compared with the high glucose group and Ad-EGFP group.

The results of immunofluorescence revealed that PRR protein expression in cardiac fibroblasts was increased under high glucose conditions compared with the normal glucose group and high permeability group (Fig. 3A,B, *p* < 0.05). The YAP protein expression level in 5 groups was also evaluated by immunofluorescence, and the results showed that compared with the normal glucose group and high permeability group, YAP protein expression was significantly upregulated in the high glucose group (Fig. 3C,D, *p* < 0.01). After Ad-PRR transfection, YAP protein expression was much higher in the Ad-PRR group than in the high glucose group and Ad-EGFP group (Fig. 3E,F, *p* < 0.01).

### YAP inhibitors alleviated CTGF and Smad protein expression and oxidative stress in vitro

**Figure 4:**
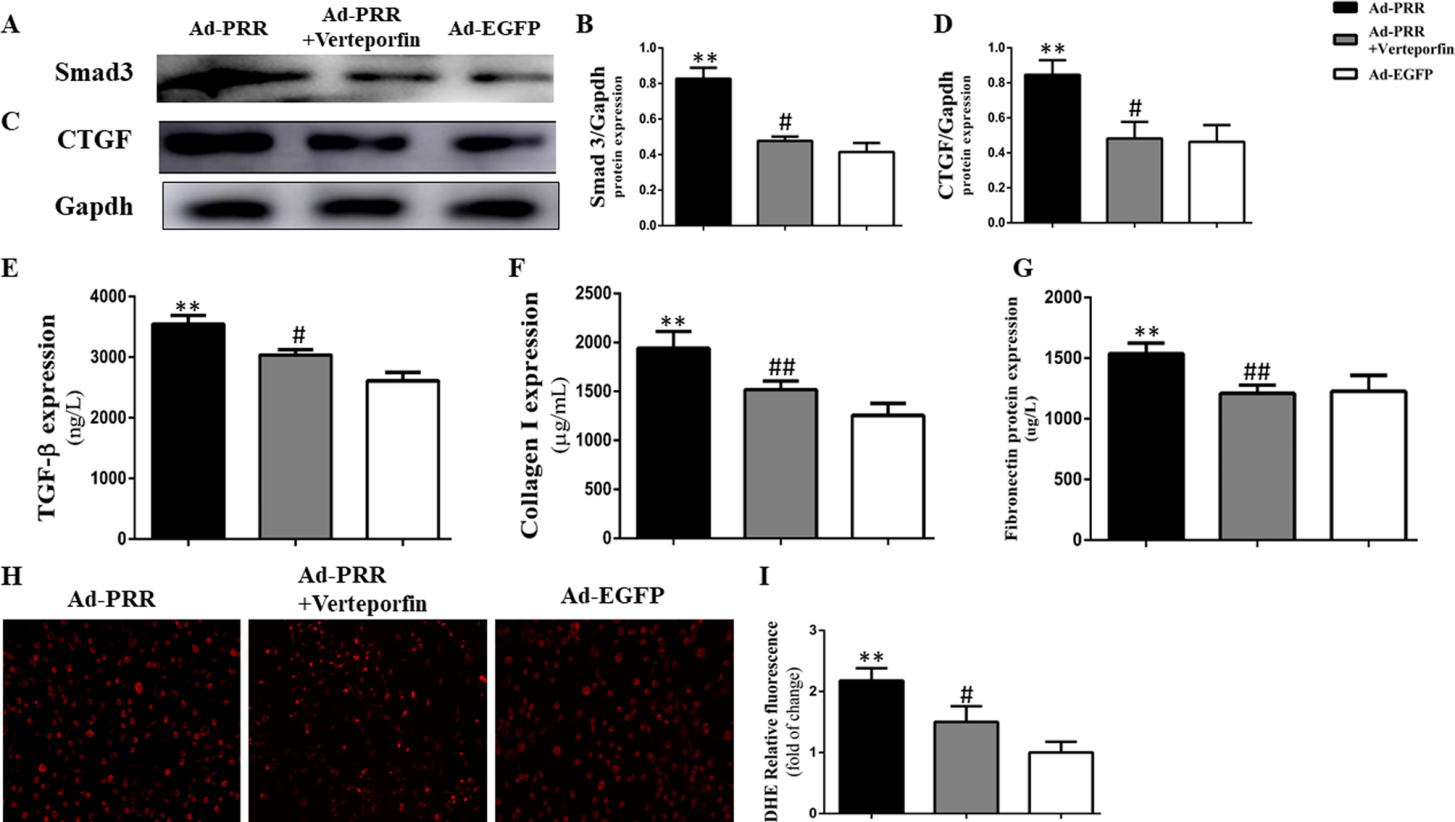
Smad3 and CTGF protein expression; TGF-β, collagen I and fibronectin expression and ROS production in cardiac fibroblasts (CFs) from the 3 groups. (**A**) Western blot showing Smad3 protein expression. Figure (**A**) is cropped picture, omitting the repeated blots from the same group. The full-length blots/gel is complemented in supplementary figure 4. (**B**) Quantitative analysis of the results shown in (**A**). (**C**) Western blot showing CTGF protein expression. (**D**) Quantitative analysis of the results shown in (**C**). (**E**–**G**) ELISA of TGF-β, collagen I, and fibronectin protein expression. (**H**) DHE showing ROS production in the 3 groups. (**I**) Quantitative analysis of the results shown in (**H**). **p < 0.01 compared with the Ad-EGFP group, #*p* < 0.05, ##*p* < 0.01 compared with the Ad-PRR group.

The results of Western blot showed that Smad3 protein expression was much higher in the Ad-PRR group than in the Ad-EGFP group, but Smad3 expression in the Ad-PRR + verteporfin group was significantly decreased (Fig. 4A,B, *p* < 0.01). Regarding the level of CTGF protein, the results also showed that CTGF protein expression was apparently attenuated in the Ad-PRR + verteporfin group compared with the Ad-PRR group (Fig. 4C,D, *p* < 0.01).

The results of ELISA showed that TGF-β protein expression was higher in the Ad-PRR group than in the Ad-EGFP group; however, the TGF-β protein expression was lower in the Ad-PRR + verteporfin group than in Ad-PRR group (Fig. 4E, *p* < 0.05). Similarly, collagen I protein expression in the Ad-PRR + verteporfin group was significantly decreased compared with that in the Ad-PRR group (Fig. 4F, *p* < 0.01). Fibronectin protein expression was also detected by ELISA, and the results showed that the administration of verteporfin could significantly downregulate fibronectin expression compared with the Ad-PRR group (Fig. 4G, *p* < 0.01).

According to the DHE results, ROS production was increased in the Ad-PRR group but was statistically lower in the Ad-PRR + verteporfin group than in the Ad-PRR group (Fig. 4H,I, *p* < 0.05).

### PRR RNAi silencing decreases PRR and YAP expression in vitro

**Figure 5:**
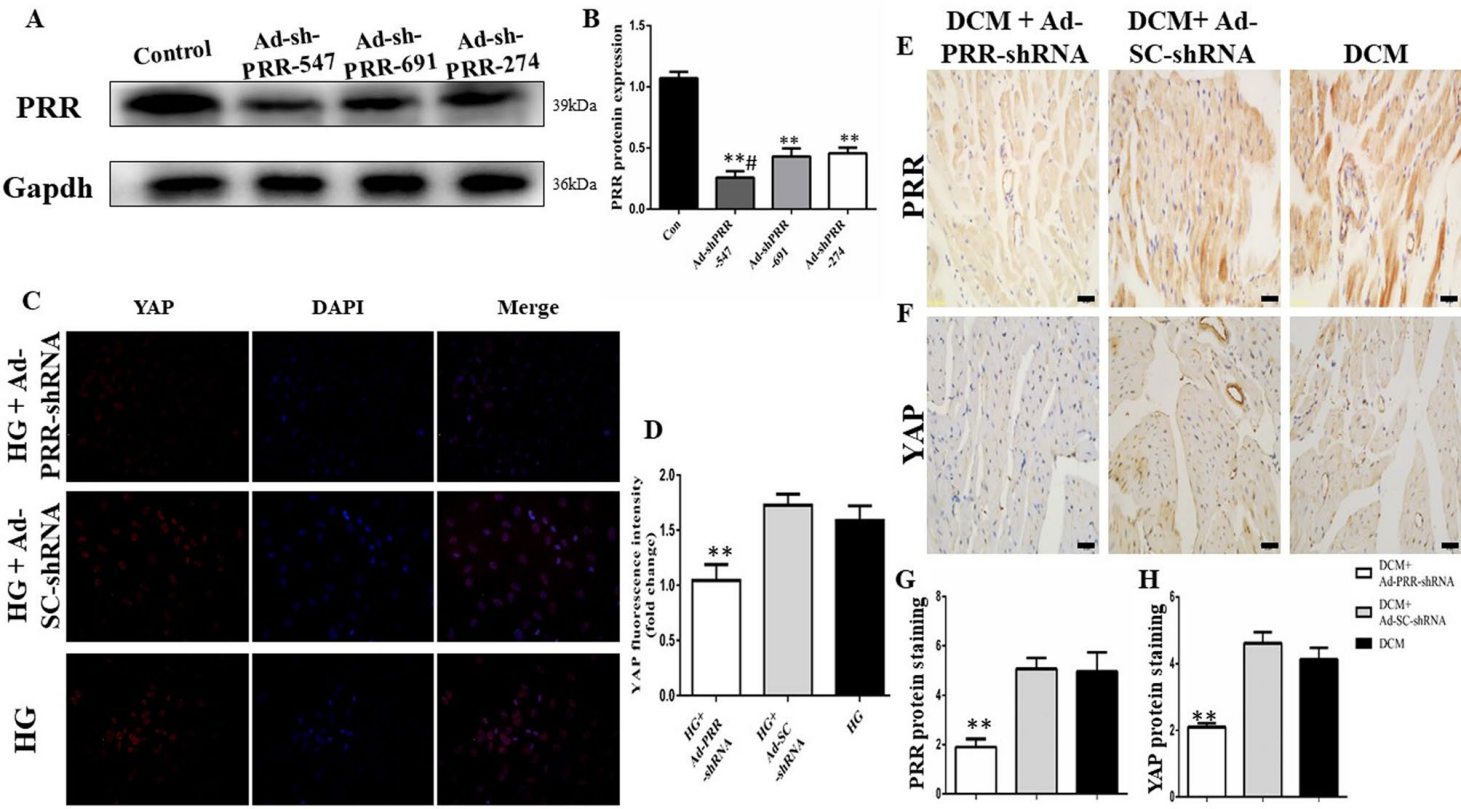
PRR and YAP protein expression, fibrosis and ROS production after PRR RNAi silencing. (**A**): Representative PRR protein expression after three types of Ad-PRR-shRNA transfection. Figure  (**A**) is cropped picture, omitting the repeated blots from the same group. The full-length blots/gel is complemented in supplementary figure 5. (**B**) Quantitative analysis of the results shown in (**A**). (**C**) Immunofluorescence showing YAP protein expression in the cardiac fibroblasts (CFs) in the high glucose group, Ad-SC-shRNA group, and Ad-PRR-shRNA group. (**D**) Quantitative analysis of the results shown in (**C**). (**E**–**H**) PRR and YAP protein expression after PRR RNAi silencing. (**E**) PRR protein expression after PRR RNAi silencing in the 3 groups. (**G**) Quantitative analysis of the results shown in (E), **p < 0.01 compared with the Ad-SC-shRNA group and DCM group. (**F**) YAP protein expression after PRR RNAi silencing in the 3 groups. (**H**) Quantitative analysis of the results shown in (**F**), **p < 0.01 compared with the Ad-SC-shRNA group and DCM group. In this part, each group had n = 20 rats at beginning, in 2 weeks took 8 rats from each group for analysis. In 4 weeks after removing dead rats, the other of rats incorporate into analysis, in DCM group had 12 rats, Ad-SC-shRNA group had 12 rats and Ad-PRR-shRNA group had 11 rats finally.

Western blot was used to determine the most efficient type of Ad-PRR-shRNA, and the results showed that the PRR protein expression in the Ad-shRNA-PRR-547 group was the lowest among the three types of Ad-PRR-shRNA (Fig. 5A,B, *p* < 0.01).

The results of immunofluorescence revealed that the YAP protein expression level was much lower in the Ad-PRR-shRNA group than in the Ad-SC-shRNA group and high glucose group (Fig. 5C,D, *p* < 0.01).

### PRR RNAi silencing attenuated myocardial PRR and YAP expression in DCM rats

The results of immunohistochemical staining showed that PRR protein expression was significantly lower with PRR RNAi silencing in the Ad-PRR-shRNA group than in the DCM group and Ad-SC-shRNA group (Fig. 5E,G, *p* < 0.01). In addition, YAP protein expression was also much lower in the Ad-PRR-shRNA group than in the DCM group and Ad-SC-shRNA group (Fig. 5F,H, *p* < 0.01).

### YAP RNAi silencing attenuated YAP and Smad3 expression and myocardial fibrosis in DCM rats

**Figure 6:**
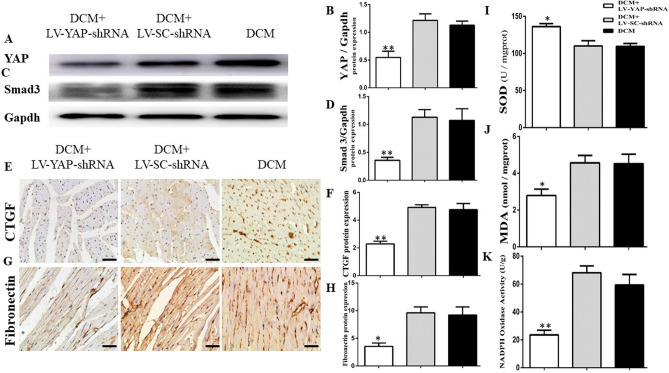
YAP, Smad3, CTGF and fibronectin expression in the 3 groups and SOD and, MDA levels and NADPH oxidase activity in the 3 groups. (**A**) YAP protein expression by Western blot. Figure (**A**) is cropped picture, omitting the repeated blots from the same group. The full-length blots/gel is complemented in supplementary figure 6. (**B**) Quantitative analysis of the results shown in (**A**). (**C**) Smad3 protein expression by Western blot. (**D**) Quantitative analysis of the results shown in (**C**). (**E**) Immunostaining of CTGF, (**F**) Quantitative analysis of the results shown in (**E**). (**G**) Immunostaining of fibronectin. (**H**) Quantitative analysis of the results shown in (**G**). (**I**) SOD levels in the 3 groups. (**J**) MDA levels in the 3 groups (**K**) NADPH oxidase activity in the DCM group, Ad-SC-shRNA group, and Ad-PRR-shRNA group. *p < 0.05, **p < 0.01 compared with the DCM group (n = 11) and LV-SC-shRNA group (n = 11). Scale bar = 20 μm, magnification × 40. In this part, each group had n = 20 rats at beginning, in 2 weeks took 8 rats from each group for analysis. In 4 weeks after removing dead rats, the other of rats incorporate into analysis, in DCM group had 11 rats, LV-SC-shRNA group had 11 rats and LV-YAP-shRNA group had 12 rats finally.

The protein expression levels of YAP and Smad3 were detected by Western blot. The results showed that the YAP protein expression in the LV-YAP-shRNA group was statistically lower than that in the DCM group and LV-SC-shRNA group (Fig. 6A,B, *p* < 0.01). The expression level of Smad3 in the LV-YAP-shRNA group was decreased compared with that in the DCM group and LV-SC-shRNA group (Fig. 6C,D, *p* < 0.01).

Furthermore, the protein expression levels of CTGF and fibronectin were detected by immunohistochemical staining. The results showed that the CTGF protein expression in the LV-YAP-shRNA group was statistically lower than that in the DCM group and LV-SC-shRNA group. (Fig. 6E,F, *p* < 0.05). Then, the results of immunohistochemical staining also showed that fibronectin protein expression was statistically lower in the LV-YAP-shRNA group than in the DCM group and LV-SC-shRNA group (Fig. 6G,H, *p* < 0.05).

### YAP RNAi silencing attenuated oxidative stress responses in DCM rats

The analysis of SOD and MDA results showed that the SOD level in the LV-YAP-shRNA group was upregulated compared with that in the DCM group and LV-SC-shRNA group (Fig. 6I, *p* < 0.05). In contrast, the MDA level in the LV-YAP-shRNA group was lower than that in the DCM group and LV-SC-shRNA group (Fig. 6J, *p* < 0.05). Similarly, YAP RNAi silencing in LV-YAP-shRNA group could obviously decrease the activity of NADPH oxidase in the LV-YAP-shRNA group compared with the DCM group and LV-SC-shRNA group (Fig. 6K, *p* < 0.01).

### Body weight and blood pressure and Blood glucose levels

Table 1 showed the body weight, blood pressure, and blood glucose in rats. All of these data are from three independently experiment parts. In each experiment part, we all set a new “DCM group” as “DCM control group” in order to eliminate other unnecessary outside disruptive factors and avoid errors, such as feeding conditions, different batches of rats et al.

**Table 1:** Body weight, blood pressure, and blood glucose in three separate experiments in rats.

Groups	Weight (g)	SP (mmHg)	DP (mmHg)	Glucose
DCM	302.3 ± 13.86	131.60 ± 4.615	82.00 ± 6.364	27.06 ± 0.48
DCM + SC-shNA	310.2 ± 17.16	133.20 ± 7.050	81.40 ± 4.393	27.34 ± 0.25
DCM + PRR-shRNA	337.6 ± 17.51	130.40 ± 4.878	79.00 ± 6.442	26.44 ± 0.32
DCM	256.06 ± 7.46	113.64 ± 3.32	79.27 ± 2.72	23.72 ± 1.79
DCM + Ad-EGFP	255.17 ± 9.87	110.18 ± 4.45	80.73 ± 2.33	24.25 ± 1.64
DCM + Ad-PRR	255.03 ± 9.31	109.55 ± 5.41	78.73 ± 3.26	27.07 ± 1.29
DCM	330.99 ± 19.28	122.25 ± 2.38	92.58 ± 2.92	27.96 ± 1.39
DCM + LV-SC-shRNA	323.36 ± 31.06	123.00 ± 5.93	91.25 ± 2.57	28.02 ± 1.33
DCM + LV-YAP-shRNA	381.51 ± 18.47	123.75 ± 5.26	91.45 ± 4.70	26.20 ± 1.51

The result showed that the body weight, systolic pressure (SP) and diastolic pressure (DP) and blood glucose levels were not statistically different among the groups in three separate experiments.

## Discussion

In this study, we found that PRR activated YAP expression and further exacerbated fibrosis and oxidative stress in both myocardial tissues and cardiac fibroblasts under high-glucose conditions. However, the administration of the YAP inhibitor verteporfin in vitro and YAP RNAi silencing in vivo could reverse these pathological changes. In contrast, inhibition of PRR expression by PRR RNAi silencing could decrease YAP expression in vivo and in vitro. Based on our results, we speculated that the PRR–YAP pathway exacerbated oxidative stress production and prompted myocardial fibrosis in DCM.

Diabetic cardiomyopathy (DCM) is characterized by a series of pathological processes, including inflammation, oxidative stress, apoptosis and myocardial fibrosis, that finally result in cardiac remodeling, cardiac dysfunction and can even lead to heart failure^2^. There is a consensus that inappropriate systemic and cardiac tissue renin-angiotensin system (RAS) activation is strongly related to the worsening of the DCM pathological process^3^.

The PRR is a newly discovered component of the RAS with multiple function and at least three intracellular forms, including the full-length protein (39 KD), an amino-terminal fragment [28KD, also known as soluble (P)RR] and a carboxy-terminal fragment (also known as M8-9).

As a multifunctional receptor, existing research has found that PRR not only exercises RAS-related function but also works as an accessory subunit of V-ATPase which is essential for cell survival^16,17^. In most pathological conditions, PRR mainly functions independently on RAS, such as MAPK signaling or Wnt/β-catenin signaling et al. It has been reported that PRR play an important pathological role and have therapeutic implications in cardiovascular disease. A recent clinical investigation showed that plasma s(P)RR levels could be a promising evaluative indicator for the severity of chronic heart failure with a reduced ejection fraction^4,18^; this means that the PRR plays a key role in cardiovascular disease. A recent study by Kim Connelly et al. found that PRR gene and protein expression were significantly increased in the hearts of diabetic rats and are related to DCM cardiac dysfunction^9^. Similarly, in our present study, the results of immunohistochemistry showed that the PRR was significantly upregulated in DCM rats, and the immunofluorescence results also demonstrated that high glucose could stimulate PRR overexpression in cardiac fibroblasts (CFs).

Previous studies suggested that “PRR” is in fact a protein with largely RAS and renin/prorenin independent functions. Some evidence indicates PRR can act in a variety of intracellular signaling cascades independently of RAS in heart and kidney, including Wnt/β-catenin signaling, NOX4 signaling, as well as, could work as an element of the vacuolar H+-ATPase independently of angiotensin II (Ang II)^19–21^. In this study, we found PRR may through PRR–YAP pathway independently of RAS and prorenin play a key role in the pathogenesis of DCM.

Yes-associated protein (YAP) is an important transcriptional cofactor, and it has been reported that nuclear-located YAP could enhance transcriptional activities of several functional proteins, such as TEAD family proteins, Smad family proteins, and CREB, and mediate fibrosis, oxidative stress and other disease pathological progress^11,12^. It is reported that YAP activity could be mediated by several ways including the RAS and MAPK pathways. Chen et al. recently observed that high glucose could activate YAP expression in the kidney^15^. As the PRR is a pivotal RAS membrane receptor demonstrated to be activated in DCM, it is reasonably believed that the PRR could also be involved in YAP mediation in DCM^22^. In our study, the results of immunohistochemical staining showed that PRR overexpression in DCM rats would exacerbate YAP expression in the myocardium; the same trend is displayed in high glucose stimulated CFs, and the results of immunofluorescence showed that upregulated PRR by Ad-PRR could further increase YAP expression under high glucose conditions. However, PRR RNAi silencing could reverse the upregulation of YAP expression in DCM in vivo and in vitro.

Myocardial fibrosis is one of the most important pathological processes in DCM that will further induce ventricular remodeling, cardiac dysfunction and even heart failure. A previous study has demonstrated that the PRR plays an important role in myocardial fibrosis associated with diseases^23^. Regarding the function of YAP in tissue fibrosis, Stephen Szeto et al. recently reported that activated YAP could trigger profibrotic activity in renal diseases. However, the YAP-specific blocker verteporfin was able to limit this negative effect^24^. In addition, Ge et al. found that the traditional Chinese medicine dihydrotanshinone I attenuated the YAP/TEAD2/CTGF pathway against the progression of hepatic fibrosis^25^. Our present study demonstrated that the PRR mediates myocardial fibrosis caused by DCM. The results showed that PRR overexpression in the myocardium significantly upregulated the expression of TGF-β, CTGF, collagen I and fibronectin in DCM rats. However, YAP RNAi silencing inhibited the production of YAP to rescue myocardial fibrosis in DCM rats. However, in in vitro experiments, YAP blockade was also effective at inhibiting the negative effects of PRR overexpression under high glucose conditions. The results by Western blot showed that the YAP blocker verteporfin was able to reduce Smad3 and CTGF expression induced by PRR overexpression. At the same time, our results showed that the administration of verteporfin could apparently alleviate TGF-β, collagen I and fibronectin expression in high glucose-stimulated CFs induced by PRR overexpression. All of these findings indicated that the PRR activated YAP and further mediated high glucose-induced myocardial fibrosis. In a word, the PRR takes part in the pathogenesis of fibrogenesis via the PRR–YAP pathway in DCM.

Although it is contrary to what Rosendahl et al. found in their study that directly constructed PRR overexpression transgenic mice didn’t show expectedly tissue fibrosis phenotype^7^. It seems not in consistent with what we found, but they also explained that this result just means excessive PRR may not a primary initiator of cardiovascular and renal damage but it could aggravate tissue damage due to inflammation or diabetes.

Next, we observed how the PRR–YAP pathway acts on DCM-induced oxidative stress. First, our study revealed that the PRR mediates oxidative stress in DCM. The results showed that PRR overexpression in DCM facilitated ROS production in vitro and promoted MDA levels and NADPH activity but downregulated SOD levels in vivo. However, downregulated YAP expression by YAP RNAi silencing yielded the opposite effects. We further demonstrated the function of the PRR–YAP pathway in oxidative stress caused by DCM. Our DHE results showed that PRR overexpression could exacerbate high glucose-induced ROS production in vitro, but administration of verteporfin inhibited this pathological progress. This means that the PRR–YAP pathway could mediate the oxidative stress caused by DCM. From our results, we concluded that the PRR–YAP pathway plays a key role in oxidative stress and myocardial fibrosis in DCM.

However, until now there is no demonstration about direct interactions between YAP and PRR. Previous study found that PI3K-PDK1 signaling inhibit Hippo pathway signaling and trigger YAP nuclear accumulation^26^. Meanwhile, some papers have reported that PRR can interact with PI3K signaling pathway, under high glucose condition, PRR siRNA significantly decreases autophagy through the PRR/PI3K/Akt/mTOR signaling pathway^27^. So, we speculated that the relationship between PRR and YAP is indirect and PRR produces YAP expression maybe through PI3K-PDK1 signaling. We are working on it.

## Conclusion

In conclusion, our study demonstrated that under high glucose conditions, the expression levels of the PRR and YAP are high in the myocardium, and alteration PRR expression could induce the same change in YAP with the same trend both in DCM rats and in high glucose-induced cardiac fibroblasts. The high expression of PRR and YAP consequently play important roles in oxidative stress and myocardial fibrosis in DCM. Furthermore, YAP blockade is able to attenuate the acceleration of myocardial fibrogenesis and the aggravation of redox abnormalities in DCM caused by PRR activation. Thus, we speculated that PRR–YAP activation could accelerate oxidative stress responses and myocardial fibrosis in DCM. YAP is an important nuclear-located transcriptional cofactor that directly regulates the expression of some necessary functional proteins such as SMAD and CTGF that are involved in DCM pathological changes, such as tissue fibrosis and oxidative stress. YAP is more likely to be an upstream signal that may play a more fundamental and stronger role in the mediation of pathological progression than other PRR-associated pathways. In conclusion, treatment targeting the PRR–YAP pathway may be a novel and more available therapeutic method for DCM.

## Materials and methods

### Adenovirus carried PRR gene construction

The amplified open reading frame (ORF) of PRR gene, which was designed by GenePharma Company (Shanghai, China), was inserted into pDC316 plasmid vector to construct pDC316-PRR shuttle plasmid. Then pDC316-PRR plasmid which contains MCMV promoter and the SV40 polyadenylation was co-transformed with adenovirus vector into the E.coli for recombinant adenoviruses (Ad) carried PRR (Ad-PRR) construction. At the same time recombinant adenoviruses control transgene EGFP (Ad-EGFP) was also constructed to be a control.

### PRR shRNA construction

PRR shRNA fragments were designed by GenePharma Company (Shanghai, China) and further to construct adenoviruses-PRR-shRNA (Ad-PRR-shRNA) and adenoviruses-scrambled shRNA (Ad-SC-shRNA). There are total three types of Ad-PRR-shRNA, including Ad-PRR-shRNA-547, Ad-PRR-shRNA-691 and Ad-PRR-shRNA-274, were designed for next selection.

H9C2 cell line was used to pick out the most efficient fragment from these three fragments in Ad-PRR-shRNA. The result was demonstrated by Western blot. The Western blot showed that Ad-PRR-shRNA-547 (sequence are GCTCCGTAATCGCCTGTTTCA for PRR) achieved the most reliable effects on PRR expression blocked.

### YAP shRNA construction

YAP shRNA fragments were designed by GENECHEM Company (Shanghai, China) and further to construct lentivirus-YAP-shRNA (LV-YAP-shRNA) and lentivirus-scrambled-shRNA (LV-SC-shRNA). The sequence is AGAGATACTTCTTAAATCA for YAP and the efficacy of lentivirus transfection was detected by Western blot.

### Experimental animals

Experimental animals were constructed by male Wistar rats, 8-week-old, weighted from 200 to 250 g. All of these animals were randomly divided into 3 parts, including PRR overexpression part, PRR RNAi silencing part to respectively detect the effects of PRR on YAP expression and pathological progress, and YAP RNAi silencing part to explore the effects of YAP on the pathological changes in DCM. Wistar rats were bought from Shandong University Animal Center and all of them were kept in an appropriate circumstance with the 22 ± 2 ℃ temperature, 55 ± 5% humidity and 12-h light and 12-h dark time cycle.

DCM models were successfully established by a single streptozotocin (STZ, dissolved in 0.1 M citrate buffer liquid, 65 mg/kg) intraperitoneal injection. One week after STZ administration, rats with blood glucose levels > 11.1 mmol/L with classical clinical symptoms were chosen for further treatment.

Rats in PRR overexpression part were divided into 4 groups control group, DCM group, Ad-PRR group and Ad-EGFP group (n = 20 per group), 12 weeks after STZ administration, The rats in the DCM group, Ad-PRR group, and Ad-EGFP group were anesthetized by 10% chloral hydrate (300 mg/kg) and mechanically ventilated with a VIP Bird ventilator (Bird Products Corp., Palm Spring, CA, USA) with a tidal volume of 3.0 ml and a respiratory rate of 60 cycles/min; the hearts were exposed after opening the pericardium. Rats in the DCM group, Ad-PRR group, and Ad-EGFP group received an intramyocardial injection of 1 × 10^9^ pfu of Ad-PRR (diluted in PBS, 200 µl), Ad-EGFP (200 µl) and phosphate-buffered saline (PBS) only, respectively, in 5 separate locations in the left ventricular free wall of the heart. After finishing all of the operations the pericardium and chest were closed, and the animals were allowed to recover from anesthesia in normal conditions. Eight rats from each of group were euthanized 2 weeks after virus transfection, and the rest were maintained until 4 weeks after it. The myocardium of the left ventricle was collected for pathological analysis.

Rats in PRR RNAi silencing part were divided into 3 groups: the DCM group, Ad-SC-shRNA group and Ad-PRR-shRNA group (n = 20 per group); each group will respectively receive intravenous injection of Ad-PRR-shRNA (1 × 10^9^pfu), Ad-SC-shRNA group (1 × 10^9^pfu) or PBS via the tail vein. Eight rats from each group were euthanized 2 weeks after virus transfection, and the rest were maintained until 4 weeks after transfection. The myocardium of the left ventricle was collected for pathological analysis. During the experiments, one rat in the DCM group, one rat in the Ad-PRR-shRNA group died.

Rats in YAP RNAi silencing part were divided into 3 groups: the DCM group, DCM + LV-SC-shRNA group and DCM + LV-YAP-shRNA group (n = 20 per group); each group will respectively receive intravenous injection of LV-SC-shRNA (1 × 10^7^pfu) for DCM + LV-SC-shRNA group. and LV-YAP-shRNA (1 × 10^7^pfu) for DCM + LV-YAP-shRNA or PBS for DCM group via the tail vein. Eight rats from each of group were euthanized 2 weeks after virus transfection, and the rest were maintained until 4 weeks after it. The myocardium of the left ventricle was collected for pathological analysis. During the experiments, one rat in the DCM group and one rat in the DCM + LV-SC-shRNA group died.

The animal protocol was in compliance with the Guide for the Care and Use of Laboratory Animals by the National Academy of Sciences and published by the US National Institutes of Health (NIH Publication No. 86-23, revised 1996) and the Principles of Laboratory Animal Care formulated by the National Society for Medical Research. The study was carried out in compliance with the ARRIVE guidelines. The protocol was approved by the Institutional Animal Care and Use Committee at Shandong University.

### Immunohistochemical staining

Myocardial tissues were divided into 2–3-cm thickness pieces and fixed in 4% paraformaldehyde at least 24 h. Each block was embedded in paraffin, and 4.5-μm-thick sections were produced. The sections were used to detect specific protein expression by immunohistochemical staining. The 4.5-μm-thick sections were deparaffinized, and antigen retrieval was performed with a temperature over 95 ℃ by 3-min high power and 13-min low power heating in a microwave oven. Goat serum for 15 min at 37 ℃ was used for nonspecific reaction blockade. Then, sections were incubated with primary polyclonal anti-PRR (Abcam, Cambridge, MA) diluent at a ratio of 1:200 with PBS, anti-YAP (Cell Signaling Technology, Danvers MA, USA) diluent at a ratio of 1:150 with PBS, anti-CTGF (Abcam, Cambridge, MA) diluent at a ratio of 1:200 with PBS, anti-TGF-β(Abcam, Cambridge, MA) diluent at a ratio of 1:150 with PBS and anti-fibronectin (Abcam, Cambridge, MA) diluent at a ratio of 1:150 with PBS overnight at 4 °C. Horseradish peroxidase-labeled anti-secondary antibody (Abcam, Cambridge, MA) was incubated for 20–30 min at 37 °C. Negative controls were free from the primary antibody. For each measure, we selected at least 6–8 serial sections from each heart for examination and analysis. Then, we compared the level of target proteins across different groups. The results were viewed under a confocal FV 1000 SPD laser scanning microscope (Olympus, Japan) to analyze the positive expression area intensity and the total size of the examined tissue area. The final result is the ratio of the positive area intensity to the total size of the examined tissue area.

### Western blot analysis

Total proteins were extracted from myocardium tissues of 6 groups, separated by SDS-PAGE and transferred to polyvinylidene difluoride (PVDF) membrane. According to the standard molecular weight marker of Prestained Color Protein Molecular Weight Marker (beyotime, China) and molecular weight of targeted protein, we divided total PVDF membrane into several parts beforehand. Each part of aforementioned PVDF membrane covered one of specific targeted protein molecular weight location, in order to make them subsequently be able to be incubated adequately by specific antibodies reagent. 5% skim milk for non-specific protein blockade for 2 h under room temperature, PVDF membranes were incubated with specific antibodies which were diluted by WB primary antibody diluent at the ratio 1:1000, overnight at 4 °C. Specific primary antibody included PRR (Abcam, Cambridge, MA), YAP (Cell Signaling Technology, Danves MA, USA), GAPDH (Abcam, Cambridge, MA), CTGF (Abcam, Cambridge, MA). 1:5000 horseradish peroxidase-conjugated secondary antibodies (Abcam, Cambridge, MA) were used to bind with specific antigen–antibody complex. Immunoreactive bands were visualized by use of an enhanced chemiluminescence reagent. Densitometry involved use of Image J.

### Malondialdehyde (MDA)

MDA contents of myocardium tissues were detected by a commercially available kit based on thiobarbituric acid (TBA) reactivity (NanJing JianCheng Bioengineering Institute, China). Briefly, after fully mixing 0.1 mL of myocardium tissue homogenate with the reaction reagent, the mixture were heated under 95 ℃ temperature for 40 min. Next, after cooling and centrifuging the mixture, a supernatant was obtained and added with TBA, and then the absorbance of 6 different groups’ red compounds were observed at 532 nm with a spectrophotometer (Thermo Fisher Scientific, USA). Other procedures were carried out following the assay kits instructions. The T-SOD activity was expressed as units/mg protein.

### Superoxide dismutase (SOD)

1% Myocardium tissue homogenate was used to detect the level of the generation of Superoxide Dismutase (SOD). Under the reaction of commercially available kit’s chromogenic agent, Nitrite produced a purple color. Absorbance of each group was detected at 550 nm with the spectrophotometer (Thermo Fisher Scientific, USA). The T-SOD activity was expressed as units/mg protein.

### NADPH oxidase activity

Myocardium tissue homogenate was produced by myocardial tissues and detected NADPH oxidase activity by NADPH oxidase assay kit (NanJing JianCheng Bioengineering Institute, China) and followed instruction to detect each group’s NADPH oxidase activity. The absorbance at 20 s and 80 s of experiments were respectively detected in 600 nm wave length.

### Cell culture and treatments

Cardiac fibroblasts from 1 to 3-day-old neonatal rats were extracted for vitro experiments. First, the whole heart was cut into pieces as small as possible; then, myocardial matrix was removed with collagenase. The samples were preincubated for 1.5 h to distinguish cardiac fibroblasts from myocardial cells because of their different abilities for wall adherence. After discarding the supernatant and adding myocardial fibroblast culture medium, cardiac fibroblasts were cultured in a humidified atmosphere of 5% CO_2_ and 95% air at 37 ℃ until they exhibited a typical growth pattern. The culture medium consisted of endotoxin-free DMEM (Thermo Fisher Scientific, USA) supplemented with 5 mM glucose and 10% FBS (Thermo Fisher Scientific, USA). The purity of cardiac fibroblasts was ensured by detection of cardiac fibroblast-specific vimentin expression. After 2–3 generation, cells were transplanted into six-well cell plates for further treatments^28^.

To determine how high glucose influences PRR and YAP expression in cardiac fibroblasts, cells were divided into 3 groups, including the normal glucose group (5.5 mM glucose), high glucose group (25 mM glucose) and mannitol high permeability control group (19.5 mM mannitol + 5.5 mM glucose), and the PRR and YAP expression levels were detected by immunofluorescence staining. A high glucose solution not only changes the sugar content but also changes the osmotic pressure in the cellular environment, which may induce side effects not associated with the experimental aims. To eliminate this side effect, we added the high permeability control group, which has the same osmotic pressure but normal glucose to show the effect of osmotic pressure on our experiments*.*

### Ad-shRNA and Ad-PRR transfection and treatments in vitro

The cardiac fibroblast count was 1 × 10^8^. The cells were transplanted into six-well plates and grown to 80–90% confluence for further treatments. Cell experiments were divided into two parts, PRR overexpression part and PRR RNAi interference part. Cells in two experiments were transfected.

### Ad-PRR transfection and treatments in vitro

First, cells were cultivated in DMEM without FBS for more than 12 h. Then, cells in the Ad-PRR group and Ad-EGFP group were transfected with Ad-PRR and Ad-EGFP, respectively. The quantity of adenovirus transfection in each well was assured with MOI values up to 150:1. After 12 h of incubation in transfection reagent, the cells were then switched to normal medium for another 12 h of recovery followed by further experiments.

Cells in the Ad-PRR + verteporfin group were pretreated with verteporfin (0.25 µM, Selleck Chemicals Company, Houston, Texas, USA) first in normal glucose culture medium for 1 h and then changed to 25 mM glucose with drugs for another 12 h.

### Ad-shRNA transfection in vitro

Cells were cultivated in DMEM without FBS for more than 12 h first. Then, cells in the Ad-SC-shRNA group and Ad-PRR-shRNA group were transfected with Ad-SC-shRNA and Ad-PRR-shRNA, respectively. The quantity of adenovirus transfection in each well was assured with MOI values up to 150:1. After 12 h of incubation in transfection reagent, the cells were then switched to normal medium for another 12 h of recovery followed by further experiments.

### Immunofluorescence staining

Cardiac fibroblasts chamber slides were used to detect PRR and YAP protein expression change in different treatments. After all of experiments PRR protein expression and YAP expression were detected by immunofluorescence staining. Cardiac fibroblasts in each group were fixed with 4% paraformaldehyde, blocked with 10% BSA and stained with anti-PRR antibody(diluted ratio 1:250, Abcam, Cambridge, MA) and YAP (diluted ratio 1:200, Cell Signaling Technology, Danves MA, USA) overnight at 4 ℃. PRR protein combined with specific primary antibody was bonded with FITC-conjugated IgG and YAP protein combined with specific primary antibody was bonded with TRITC-conjugated IgG. Cell nucleus were stained with 6-diamidino-2-phenylindole (DEPI) and samples were sealed with anti-fluorescence quenching agent (Abcam, Cambridge, MA). Immunofluorescence intensity was visualized under a Leica fluorescence microscope.

### Enzyme linked immunosorbent assay (ELISA)

Cell supernatant of each group was collected for ELISA to respectively detect the expression of type I collagen, transforming growth factor-β (TGF-β) and fibronectin in different groups. All measurement processes were followed the manufacturers' instructions of ELISA Kit (Abcam, Cambridge, MA). All the detected samples were repeated three times. OD value of the sample was determined at 450 nm wavelength by Enzyme Standard Instrument. Relative factors’ concentrations were calculated according to standard samples’ OD values and concentrations.

### Measurement of ROS

ROS level in each group was measured by oxidant-sensitive fluorescence probe DHE (Sigma-Aldrich, St. Louis, MO). Cells were cleaned by serum-free medium three times and then incubated with DHE (5uM) for 30 min. After all operations, images were captured at Excitation/Emission wavelength (535/610 nm). The fluorenscence intensity was quantified using Imagine Plus Pro analysis software in a blinded manner.

### Statistical analysis

Statistical analyses were performed using SPSS19.0 software. In each experiment, the sample size of each group is greater than 5, and the mean ± standard deviation of each indicator for each group were calculated. The differences were analyzed by one-way ANOVA. A *p* value < 0.05 was considered significant.

## Supplementary Information

Supplementary Information.
