# Supplementary material for: (Pro)renin receptor involves in myocardial fibrosis and oxidative stress in diabetic cardiomyopathy via the PRR–YAP pathway
Source: Sci Rep. 2021 Feb 5;11:3259. doi: 10.1038/s41598-021-82776-2 (PMC7865047; doi:10.1038/s41598-021-82776-2)

(Pro)renin receptor involves in myocardial fibrosis and oxidative stress in diabetic cardiomyopathy via the PRR-YAP pathway

Shiran Yu[1,4,7#], Xuefei Dong[1,2,7#], Min Yang[1,3#], Qingtao Yu[1], Jie Xiong[1], Jing Chen[5,6], Bo Dong[1,7,8]*, Qing Su[9]*

supplementary information file

Figure 1

Original images (un-cropped images)

PRR

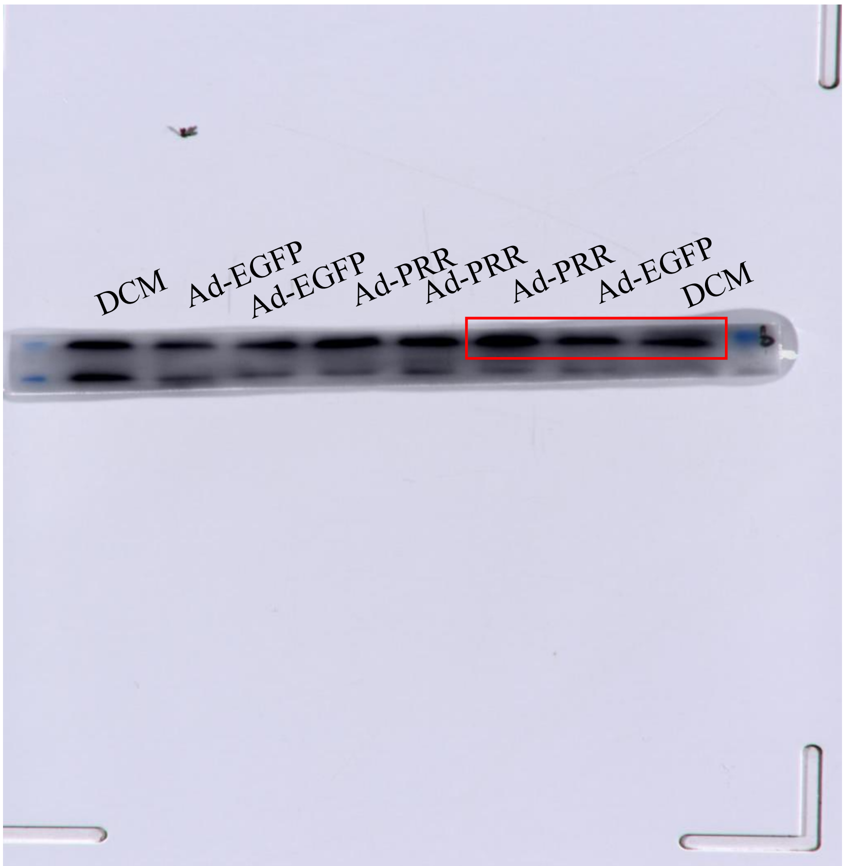

GAPDH

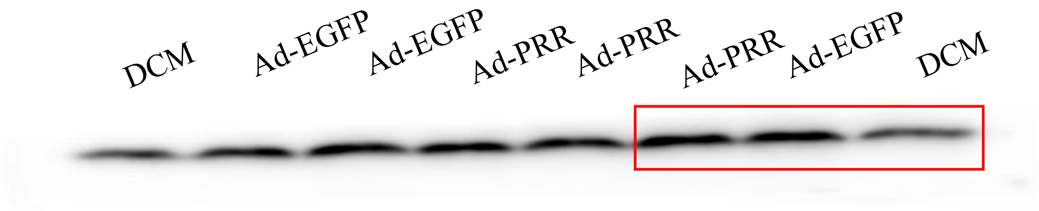

Figure 4

Original images (un-cropped images)

Smad3

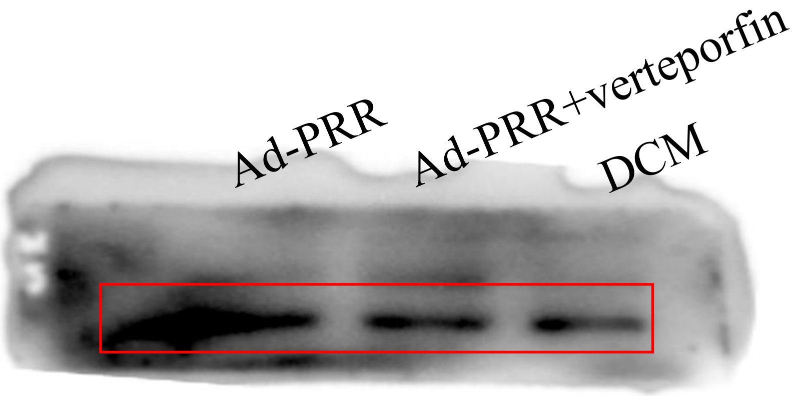

CTGF

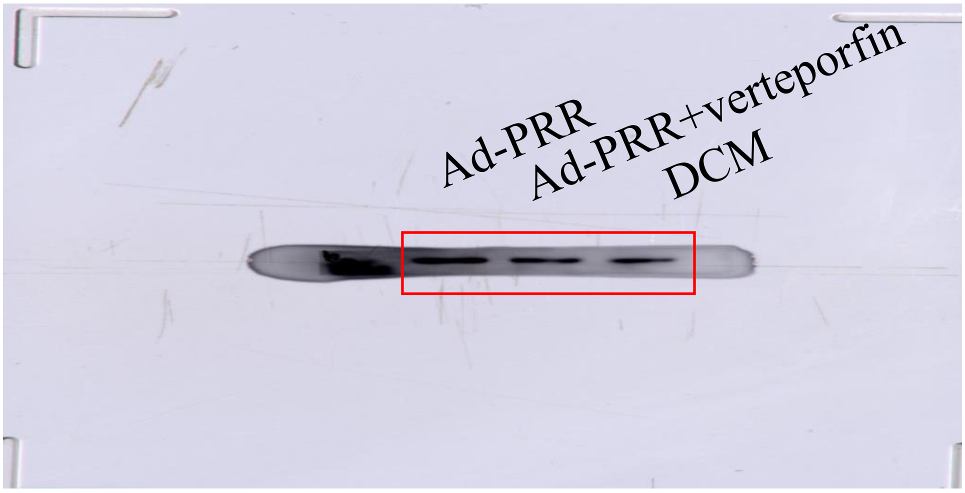

GAPDH

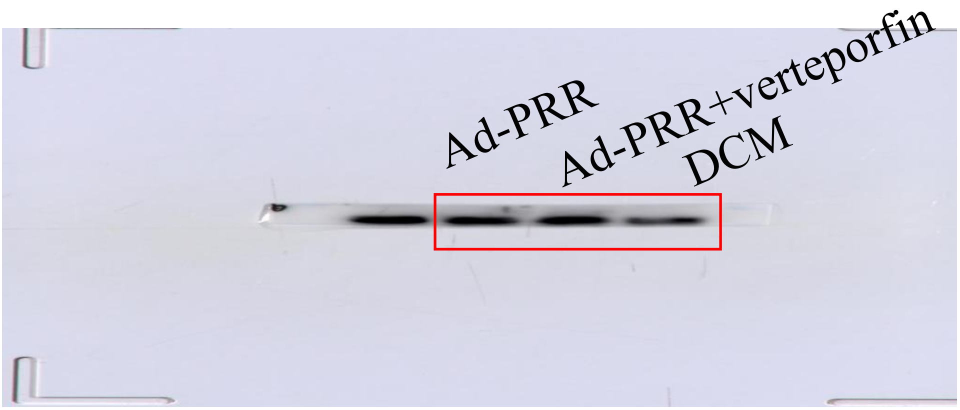

Figure 5

Original images (un-cropped images)

PRR

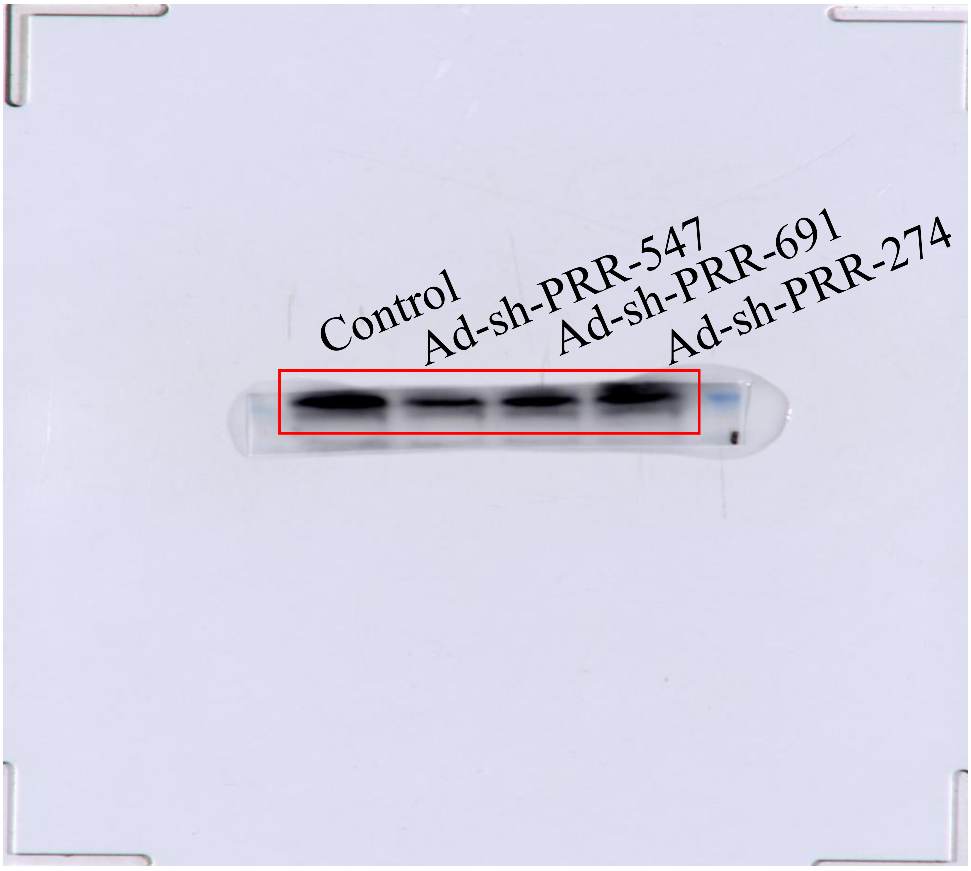

GAPDH

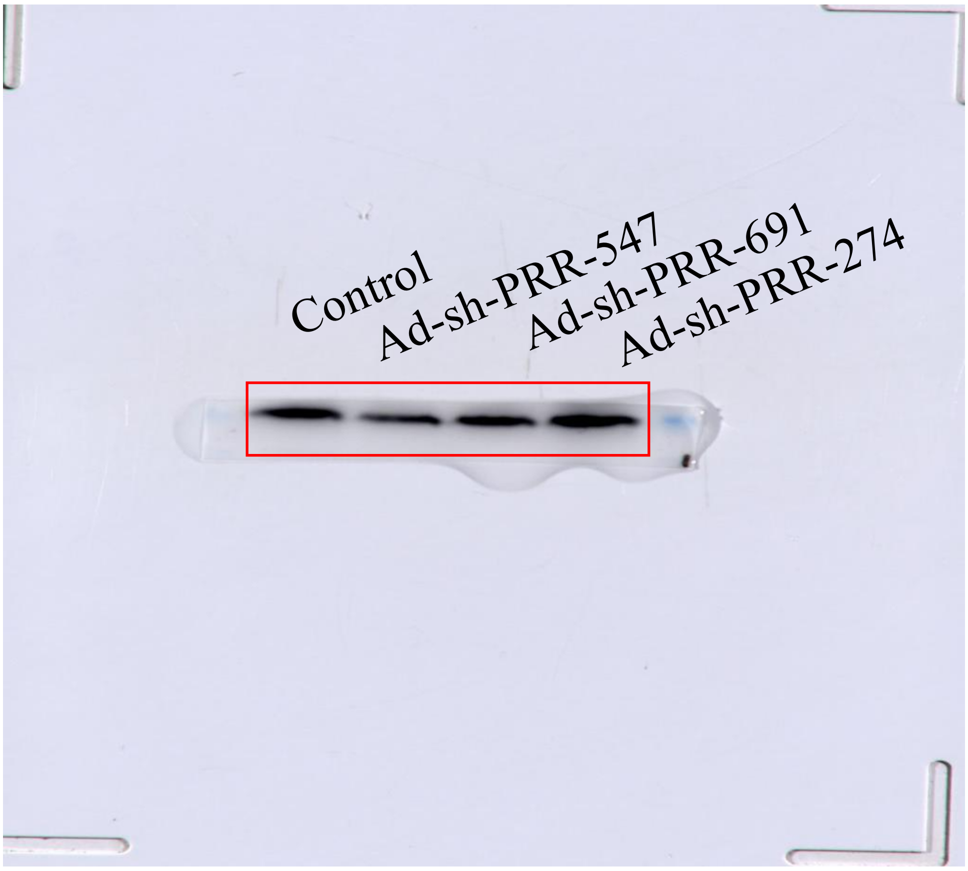

Figure 6

Original images (un-cropped images)

YAP

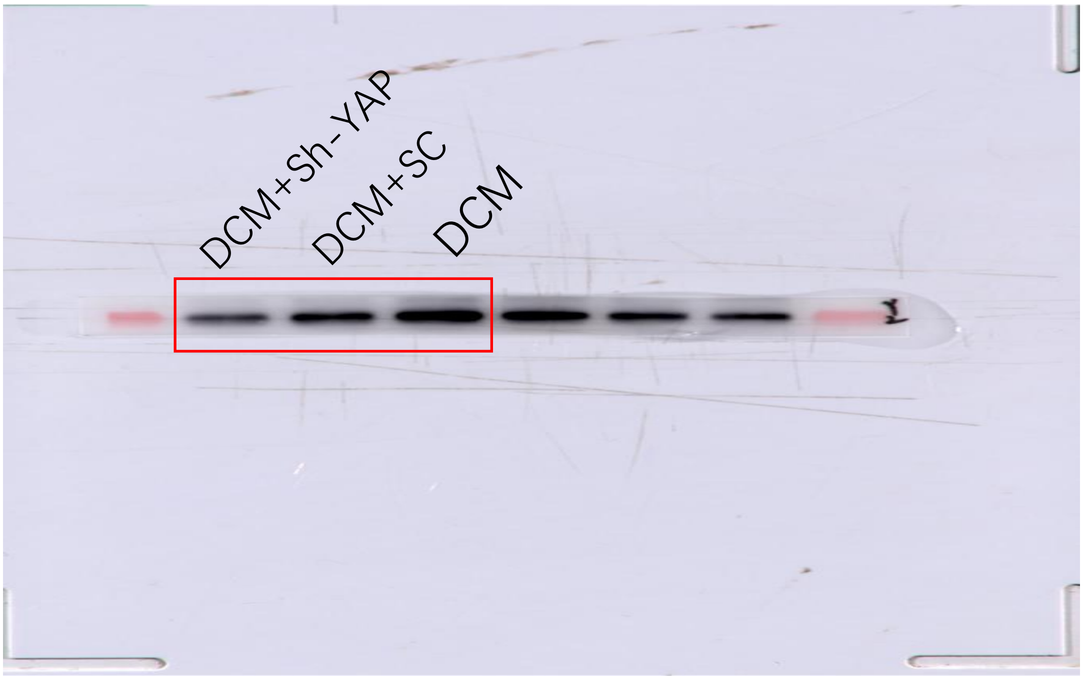

Smad3

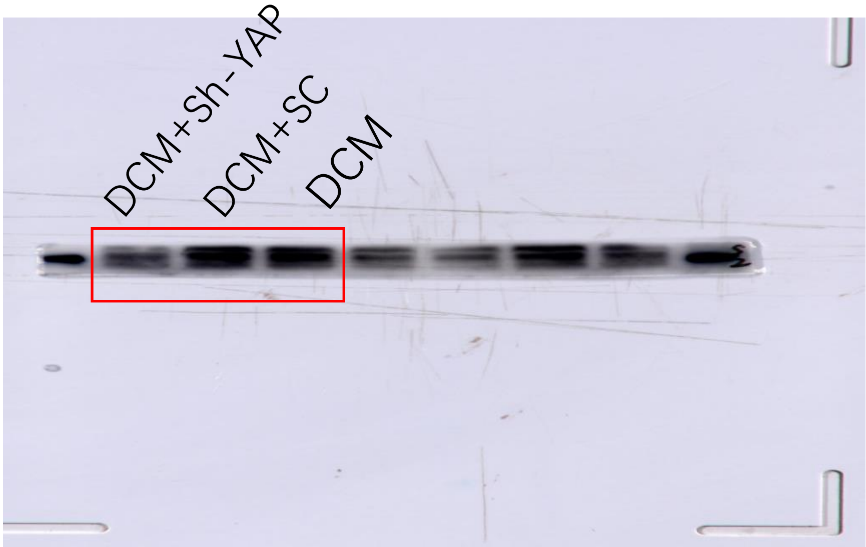

GAPDH

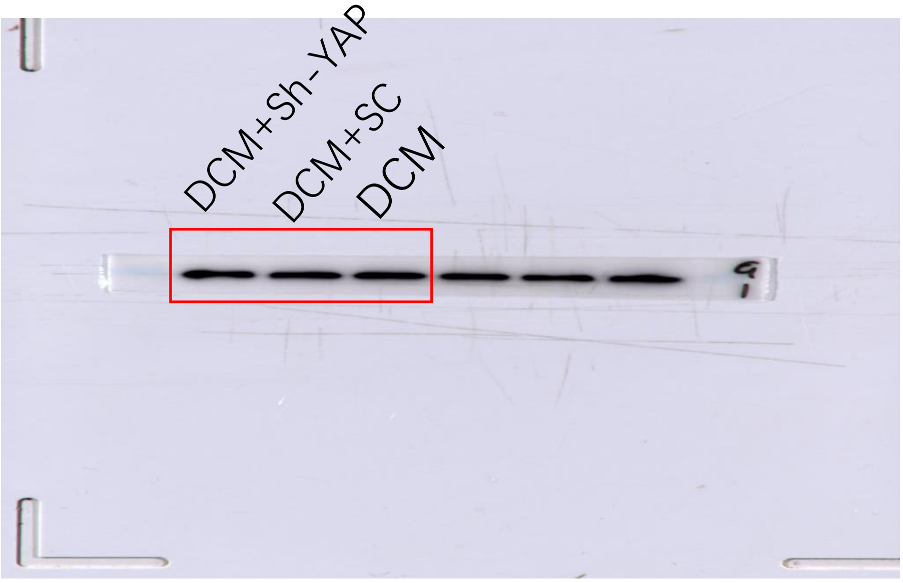

Supplement: Supplementary file 1 — Supplementary Information. [file 41598_2021_82776_MOESM1_ESM.pdf]
